# An overview of the irreversible electroporation for the treatment of liver metastases: When to use it

**DOI:** 10.3389/fonc.2022.943176

**Published:** 2022-09-01

**Authors:** Maria Paola Belfiore, Marco De Chiara, Alfonso Reginelli, Alfredo Clemente, Fabrizio Urraro, Roberto Grassi, Giuseppe Belfiore, Salvatore Cappabianca

**Affiliations:** ^1^ Division of Radiology, Department of Precision Medicine, University of Campania “Luigi Vanvitelli”, Napoli, Italy; ^2^ Department of Diagnostic Imaging, Nursing home L.Cobellis, Vallo della Lucania Salerno, Italy

**Keywords:** irreversible electroporation, ire, CT guided, liver metastases, interventional radiology

## Abstract

Tumour ablation is an established therapy for local treatment of liver metastases and hepatocellular carcinoma. Most commonly two different kind of thermic ablation, radiofrequency ablation and microwave ablation, are used in clinical practice. The aim of both is to induce thermic damage to the malignant cells in order to obtain coagulative necrosis of the neoplastic lesions. Our main concerns about these procedures are the collateral thermic damage to adjacent structures and heat-sink effect. Irreversible electroporation (IRE) is a recently developed, non-thermal ablation procedure which works applying short pulses of direct current that generate an electric field in the lesion area. The electric field increase the transmembrane potential, changing its permeability to ions.Irreversible electroporation does not generate heat, giving the chance to avoid the heat-sink effect and opening the path to a better treatment of all the lesions located in close proximity to big vessels and bile ducts. Electric fields produced by the IRE may affect endothelial cells and cholangiocytes but they spare the collagen matrix, preserving re-epithelization process as well as the function of the damaged structures. Purpose of the authors is to identify the different scenarios where CT-guided percutaneous IRE of the liver should be preferred to other ablative techniques and why.

## Introduction

Liver cancer is the third cause of oncologic-related death worldwide (1), with liver metastases being the most common form of liver involving tumour. Mortality rates of this pathological condition have seen an unprecedented increment over the last few years (2). Unluckily, only 20% to 30% of patients are eligible for surgical resection at their diagnosis; therefore, numerous alternative procedures have been developed through the years (3). Today, tumour ablation is an established therapy for local treatment of liver metastases and hepatocellular carcinoma (HCC) (4), extremely efficient with metastatic lesions smaller than 3 cm, where no outcome difference between ablation and liver resection can be seen (5). The two most common kinds of thermal ablation, radiofrequency ablation (RFA) and microwave ablation (MWA), are used in clinical practice. The aim of both is to induce thermal damage to the malignant cells in order to obtain coagulative necrosis of the neoplastic lesions. Our main concern about these procedures is the collateral thermal damage to adjacent structures, in the first place, which may involve bowel, vessels, or bile ducts and, at the same time, the heat-sink effect. The heat-sink effect is the name physicians use to refer to a cooling phenomenon due to a large vessel’s proximity (less than 1 cm), directly related to flowing blood that reduces the heat and, because of that, the ablation volume as well (6). Irreversible electroporation (IRE) is a recently developed, non-thermal, ablation procedure that works by applying short pulses of direct current that generates an electric field in the selected area. The electric field increases the transmembrane potential, changing its permeability to ions. Different theories have been proposed to explain this phenomenon, none of which seems to suit perfectly the physical and biological changes we have found so far; however, inducing nanopores throughout the cell membrane, allowing the interstitial ions to move according to their concentration gradient from the surrounding solution, seems to be the most appealing one. As a consequence of those changes, alteration of cell homeostasis develops, and finally, cell death occurs (7).

IRE was firstly developed to manage unresectable, highly vascularized, pancreatic neoplasm not eligible for common thermal ablation. Scientific data collected so far show discrete rates of success and high levels of safety. As years went by, this technique was applied to other organs such as the liver and prostate to obtain complete ablation of all those tumours that cannot be treated surgically or removed using heat-generating techniques. Despite a general lack of evidence, the data collected by previous studies showed encouraging results.

Irreversible electroporation does not generate heat, giving the chance to avoid the heat-sink effect and opening the path to better treatment of all the lesions located in close proximity to big vessels and bile ducts (8). Electric fields produced by the IRE may affect endothelial cells and cholangiocytes, but they spare the collagen matrix, preserving the re-epithelization process as well as the function of the damaged structures (9). The purpose of the authors was to identify the different scenarios where CT-guided percutaneous IRE of the liver should be preferred to other ablative techniques and why.

## Technique

**Figure 1:**
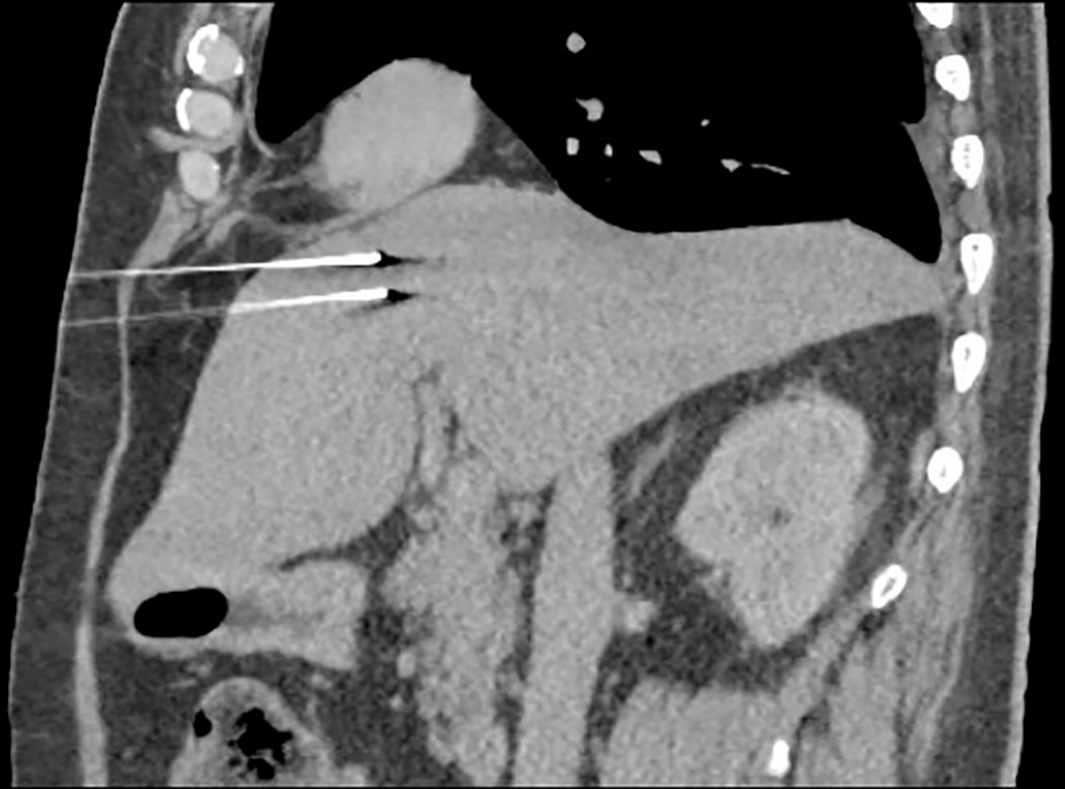
Probe position evaluation performed on CT scan. The current is directed from one probe to the other.

Better comprehension of tumour biology and the steady progress in radiology is allowing physicians to increase the number of cancer diagnosis while providing useful non-invasive treatment (10). Irreversible electroporation is a percutaneous or, less commonly, laparoscopic procedure that requires general anaesthesia and neuromuscular blocking agents. The underlying idea is to prevent involuntary muscle contraction that can accidentally arise from the electrical stimulation induced by the procedure on the motor neurons. Different kinds of plates and electrodes have been used through the years, but two to six parallel needle electrodes (Ø~1 mm) are mostly employed nowadays. After the insertion of the probes, their position is evaluated by CT imaging, and when all the electrodes are located correctly, 50 to 100 electric pulses are sequentially delivered (**Figure 1**). To mitigate the risk of arrhythmia, IRE is ECG-synchronized with the absolute refractory period of myocardial cells (11). To induce cell death, IRE needs to produce an electrical field strong enough to permanently disable the target cells homeostasis; to do so, an electric field of 300–1,000 V/cm is mandatory (12). The lethal threshold may vary according to the tissue susceptibility, but this limits decrease as more pulses are applied, eventually saturating if too many pulses are provided. Despite that, the temperature rising due to the minimal Joule effect related to the procedure increases the conductivity by 1%–3% for every Celsius degree, which may lead to a greater volume of ablation (13). Blood samples are taken before the procedure, looking for alteration in alkaline phosphatase (normal value 45–117 U/L) and bilirubin (normal value 0.2–1.0 mg/dl) levels. Due to the malignant neoplasm affecting the liver when an IRE is performed, abnormal values are not to be considered contraindications to the treatment.

Common radiofrequency thermal ablation is due to an alternating electric current, providing frictional heat directly related to the current’s intensity and duration. While the main application is for unresectable tumours, RFA provides better results when applied to masses smaller than 5 cm and even better if the treated area is smaller than 3 cm, the size of the ablation is limited, and the possibility of an incomplete resection increases with larger tumours (14). Microwave ablation allows for a flexible approach to liver tumours, and it is usually performed under conscious sedation even though general anaesthesia may be required if procedural pain is problematic. Electromagnetic microwaves agitate water molecules in the surrounding area, providing higher temperature if compared with other ablation techniques; the result is the ability to treat larger areas of affected parenchyma with better long-term outcomes (15).

Trans-arterial chemoembolization (TACE) is an alternative treatment for patients diagnosed with a primary or secondary hepatic tumour. The best candidates are asymptomatic patients without underlying liver disease with no evidence of extrahepatic spread or vascular invasion. TACE has been performed using lipiodol chemotherapy followed by embolization with gel foam particles. The major problems are the lack of safety and the relatively low rates of success, meaning that most patients experienced an incomplete tumour embolization or a relatively fast (2 years) relapse of the disease, which is why this technique is steadily being replaced by ablation (16) (**Table 1**).

**Table 1:** Comparative scheme of the different types of ablation.

Ablative modality	Principles	Indications	Advantages	Limitations
RFA	Application of electrical currents *via* an electrode, resulting in resistive heating and therefore tissue hyperthermia	BCLC O, A, B Tumour < 3 cm HCC	Most extensively studied ablation technique, broad clinical experience	Not efficient for tumour >3 cm Not subcapsular peri-vascular or adjacent to gallbladder/diaphragm
MWA	Application of propagating microwave energy in order to induce tissue hyperthermia *via* dielectric hysteresis	BCLC O, A, B Similar profile to RFA Tumour ≤5 cm	Less heat-sink effect and shorter duration of therapy compared to RFA Efficient in tumour volumes ≤5 cm	Reduced efficacy in tumours >5 cm Treatment effect varies between different vendor/device
Cryo	Gas pressures changes resulting in cooling of a cryoprobe in direct contact with tumour, resulting in fast ice crystal formation and osmotic shock	Only limited role in HCC treatment today	Well tolerated; less pain during ablation Ablation processes can be monitored effectively	High overall complication rate, such as cold shock, decreased platelet count, and bleeding Insufficiently supported by clinical studies
IRE	Alteration of transmembrane potentials to induce irreversible disruption of cell membrane integrity	Perivascular locations Applicable in peribiliary locations	No heat-sink effect Recommended in perivascular locations Preservation of the extracellular matrix	Insertion of several needles sometimes necessary Limited evidence and general lack of experience Requires general anaesthesia

RFA, radiofrequency ablation; MWA, microwave ablation; Cryo, cryoablation; IRE, irreversible electroporation; BCLC, Barcelona Clinic Liver Cancer; HCC, hepatocellular carcinoma.

Target volume evaluation after IRE is conducted by ultrasound (US) or CT (17) with US sensitivity widely ranging from 20% to 84% (18), failing to establish itself as a reliable and reproducible method while, at the same time, CT is holding such a low soft tissue contrast capacity before contrast enhancement, which is not considered a feasible solution for real-time evaluation. Magnetic resonance imaging is generally more accurate in detecting liver tumours and shows an overwhelming superiority if compared to other imaging techniques when it comes to smaller lesions. In the end, regardless of the method, radiological imaging may depict the morphological changes with great accuracy only when contrast agents are supplied, failing to assess the metabolic alterations immediately occurring in the treated area (19). Therapeutic response is mainly seen as a reduction of the neoplasm size but is not immediately visible (20). Due to its ability to detect viable tumours, fluorine-18 fluorodeoxyglucose (^18^F-FDG) PET was proposed as an imaging assay to catch early clinical response (21) (**Table 2**). While this could predict therapeutic outcomes and allow oncologists to plan new, patient-tailored, strategies, this technique failed to come into everyday clinical practice (26). Due to its minimal heat induction and its resistance to the heat-sink effect, IRE seems to be the best option to treat metastatic lesions located near proteinaceous structures in the liver such as vessels or bile ducts, showing a pivotal advantage over classical thermal ablation strategies (27). Another common indication for IRE resection includes stage III pancreatic cancer, even though stages I, II, and IV are not eligible for this kind of treatment. Renal cell carcinoma or melanoma metastases could benefit from irreversible electroporation, as well as a local recurrent disease without any radiological sign of distant metastases. Early (<6 months) local recurrence may also be treated with IRE if the dimensional increase is below 20% and the tumour size is still within the limit for electroporation treatment (28).

**Table 2:** Irreversible electroporation in liver.

Author, year of publication, reference number	No. of lesions	Age	Type of lesions	Primary efficacy [60]
Thomson et al., 2011 (22)	63	45	HCC (17), CRLM (15), other (31)	51.6
Kingham et al., 2012 (23)	65	51	HCC (2), CRLM (21), other (5)	93.8
Narayanan et al., 2014 (24)	100	54	HCC (35), CRLM (20), other (5)	NS
Niessen et al., 2017 (25)	103	64	HCC (31), CRLM (16), other (10)	68.3

HCC, hepatocellular carcinoma; CRLM, colorectal liver metastasis.

## Histological findings

In a recent study, Zhang et al. (27) took notes of the histological changes in rat liver after IRE was performed. Immediately after the procedure, no change was visible in the treated tissue, demonstrating that irreversible electroporation does not cause acute cell destruction. Three hours after IRE, the sinusoid experienced vascular congestion, while no changes in the larger structures, like bigger vessels and main bile ducts, could be seen. A clear histological difference between treated tissue and untreated areas may arise 6 h after the electroporation, where only pyknotic nuclei and neutrophil infiltration could be found in the treated region. Normal hepatocytes may be detected in the treated zone 24 h after the electroporation. The main hypothesis is that those new cells may be taken there by the blood supply from patent vessels. For some unknown reason, Kupffer cells, involved in both apoptosis and necrosis, were prominent at the site of electroporation; however, this procedure does not seem to induce apoptosis, while pyroptosis, karyorrhexis, and necroptosis are commonly observed instead (29).

The extracellular matrix remained undamaged after hepatocyte death, confirming that there was no extracellular protein damage linked to the procedure. The collagen scaffold helps the regrowth of normal parenchymal cells and may be used, in a close future, for experimental exogenous cell implantation (24).

## Complications

Though irreversible electroporation is generally considered safe, complications arise in almost 16% of the patients. The majority of these complications are directly related to the puncture itself, but uncommon side effects such as bile duct stenosis are observed in as much as 6% of the patients (30); similar rates have been reported for portal or hepatic veins stenosis or thrombosis, which still are to be considered rare (31). On rare occasions, tumour seeding through the needle tract was speculated (32). Other severe complications include intraoperative arrhythmia and atrial fibrillation, linked to the electrical pulses, postoperative portal vein thrombosis, linked with debris clothing, and pneumothorax. The mortality rate from these side effects is as low as 3% and only if no treatment is performed. Most dangerous cardiac rhythm disturbances occurred during the ablation of a big size hepatic tumour, mostly located directly beneath the diaphragm, relatively close to the inferior cardiac border (33). Despite that, IRE still stands out as the most effective method to treat unresectable liver peripherical metastasis that is located close to the diaphragm once a proper cardiac synchronization is made.

Next to those severe and uncommon complications, there are others often encountered in clinical practice; those common complications include abdominal pain, flank pain, and extrasystole. These less serious adverse effects usually completely resolve in 30 days even if no treatment is provided (23). Since we know so little about IRE, and the literature is still moving its first steps in properly explaining and exploring this new technique, from time to time, case report studies show some extremely rare adverse effects, which may be explained, to a certain extent, remembering that although IRE is a non-thermal ablation method, a little Joule effect is still theoretically possible (34).

Sporadic cases of severe portal vein stricture may be found; when this happens, the blood supply is reduced enough to require a stent placement. This device commonly leads to further complications and more interventions (24). Few studies have shown liver vessel damage after irreversible electroporation even if they all fail to assess the incidence of occurrence and underlying mechanism; therefore, the exact meaning of this rare side effect from a pathological point of view is still being debated (35). Reduced vessel patency is not immediately dangerous by itself but is strictly related to a fast deterioration in liver function, since patients undergoing IRE are usually already having chemotherapy, and a liver insufficiency could cause a sudden cessation of the treatment plan. The cause of small vessel damage after IRE is still unknown in humans, but several tests performed on laboratory animals showed oedema as being responsible for transient luminal narrowing, which usually resolves in 8 weeks (36). Some studies have supposed thermal damage due to direct contact between the plate and the vessel or post-procedural parenchymal scarring may determine sinusoid occlusion. The evidence we have today is still insufficient to determine with confidence if those conditions play a role in vascular damage or occlusion (37, 38). Incomplete or partial ablation is a typical downside in IRE, and it occurs in almost 19% of the cases mainly because of the location of the neoplasm: large bile ducts or bowel may interfere with the electrode placement, causing the procedure to be more demanding and less precise (39).

## Review

**Figure 2:**
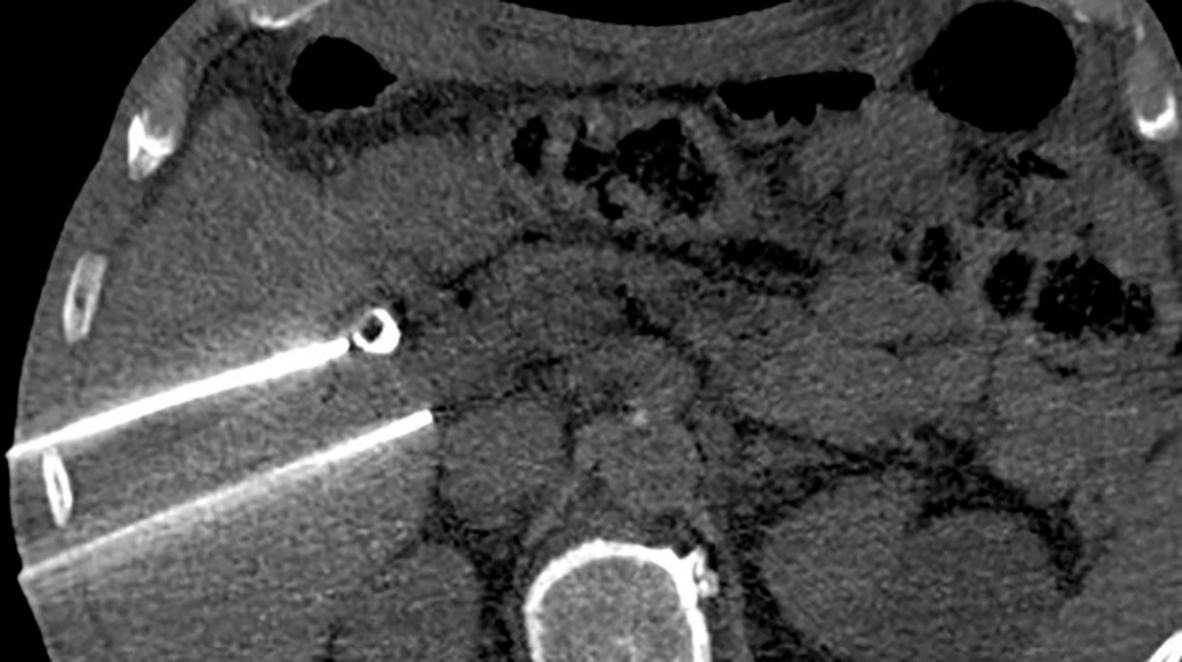
Irreversible electroporation applied near a biliary stent. Metallic devices were at first considered absolute contraindications for this kind of procedure.

When to use IRE to take advantage of its strategic role in metastases ablation is still an object of debate, mainly because the operator dependence on this procedure leads to a general lack of peer-reviewed evidence establishing a precise success rate. Moreover, patients with metastatic cancer show a wide range of co-morbidities, making it more complex to assess the exact impact of the irreversible electroporation on their general outcome (40). While tumour control was above than average for primary lesions of the liver, metastasis treated with IRE showed a poorer response, determining up to 28% of recurrence in the first 3 months after electroporation with tumours smaller than 3 cm showing a lower recurrence rate (<19%) (22). Radiofrequency ablation and microwave ablation play a pivotal role in the treatment of unresectable tumours because these techniques are safe, effective, and highly standardized, providing good outcomes with few adverse effects. IRE, however, requires the placing of more electrodes and a more accurate anatomical study. Its main role is to be performed near the big vessel and main bile ducts, and it is relatively recent when compared to other ablation techniques; therefore, among surgeons and radiologists, there is a general lack of expertise so far. Previous studies provide useful information, reporting cell death arising within the first 3 h after the treatment (23), while further investigations showed that IRE was suitable for metastases ablation even if the lesion was located near one of the main hepatic vessels, providing a fast and effective alternative to thermal ablation (40–44). Liver biomarkers and blood levels seem to be affected by this minimally invasive procedure; in fact, a modest rise can be revealed by a blood sample taken in the first 2 days after the procedure. However, since a large group of hepatocytes are being killed during the procedure, such augmentation is to be expected and showed no correlation to permanent liver damage (28). Six months of life expectancy after treatment is proven similar in both IRE and thermal ablation with the electroporation far more easily tolerated in patients with a compromised liver, which also experienced shorter hospitalization time and lower rates of re-admission (25). These findings remain valid for small liver masses up to 3 cm; bigger lesions show poorer response to non-invasive treatment and may be more efficiently approached with classical surgical techniques to the point that metastatic tumours bigger than 5 cm are hardly affected by irreversible electroporation (45). For this reason, big masses are included as current contraindications for irreversible electroporation. Metallic implants located near the procedure site aroused some hesitation regarding whether to perform the procedure, and to date, no concrete evidence can determine if they should or should not be considered absolute contraindications (**Figure 2**). Former studies pointed out those implants as responsible for affecting progression-free survival even though the exact mechanism was never totally understood. Today, more recent studies seem to be more indulgent about the mortality and morbidity outcomes in this category of patients; more investigations are thus mandatory (**Table 3**). An immediate CT scan after the procedure may detect rim enhancement in the ablated area, but said enhancement disappears 1 month later when follow-up is performed. No enhancement detected from the lesion site is the main indication of successful electroporation (46). Association with chemotherapy is an established alternative to classical IRE that significantly increases the treatment response in comparison with cytostatic agents alone such as bleomycin or cisplatin. The combined local and systemic treatment reduces the relapse risk (10.6%) and improves the life quality of our patients when chemotherapy is performed after IRE. It is postulated, incidentally, that electroporation should be performed as soon as possible after medical treatment because, due to its effect on membrane permeability, electroporation grants a higher intracellular concentration of cytostatic drugs. Unfortunately, IRE and chemotherapy association is usually reserved for palliative treatment in patients with unresectable tumours where a surgical approach is forbidden; therefore, no overall outcome impact is usually seen. This combination, however, may greatly affect the survival rate of patients with skin or prostate cancer. It is thought that the cell damage due to electrophoresis may induce the release of tumour-specific antigens, allowing the patients’ immune system to target the tumour area and complete the task of killing neoplastic cells; if this would be proven true, it could probably explain why IRE performed in nude mice is less effective (23). An interesting experiment investigating this path provides transplantation, on mice, of two different tumours in two different sites. When one nodule was treated with IRE and chemotherapy, this induced the healing of the other, untreated, nodule (24). Is it notable that among all the studies the author encountered, only one pointed out that transient elevation of pancreatic amylase may happen. However, this rare occurrence seems to be self-limiting within 48 h (24).

**Table 3:** IRE contraindication.

Absolute contraindication	Relative contraindication	No contraindication
*Cardiac*	*Cardiac*	*Cardiac*
Cardiac arrhythmias	Active coronary artery disease	History of coronary artery disease
Pacemaker	Congestive heart failure NYHA Class 2 and/or Class 3	
Congestive heart failure NYHA Class 4	· Atrial fibrillation	
*Other*	*Other*	*Other*
Severe ascites	Non-iatrogenic coagulation disorder	Epilepsy
	Moderate ascites	Minimal ascites

IRE, irreversible electroporation; NYHA, New York Heart Association.

Thomson et al. were able to avoid vessel or bile duct damage in the totality of their patients; at the same time they pointed out that, despite the anaesthesia, muscle contraction is still possible. Physicians should be aware that a correct electrode positioning is therefore mandatory since a correctly placed electrode simply does not change its place despite muscle contractions. However, IRE needles are less used and therefore less sophisticated than other needles such as the ones for MWA or RFA. Therefore, an accurate placing can be challenging. Repeated attempts may lead to subcapsular haematoma due to capsular puncture. This minor side effect is strictly dependent on the doctor’s experience and will be less common as soon as the procedure will be performed more often (22). According to Kingham et al., IRE could potentially be game-changing when it comes to near-vessel lesions, providing a new therapeutic approach. Complication rates are the same as those of other kinds of ablation therapy such as MWA or RFA, but the outcome is promising, with an initial response rate above 98%. What is new in this paper is that the ablation procedure was performed in each liver segment, but no significant difference in success rate or complication frequency could be proven (23). Another study found that vascular side effects were involving only venous structures and portal veins in particular.

Despite promising results on both human and murine models, long-term effects of IRE are still mostly unknown, and further investigation is mandatory (25).

## Conclusions

**Figure 3:**
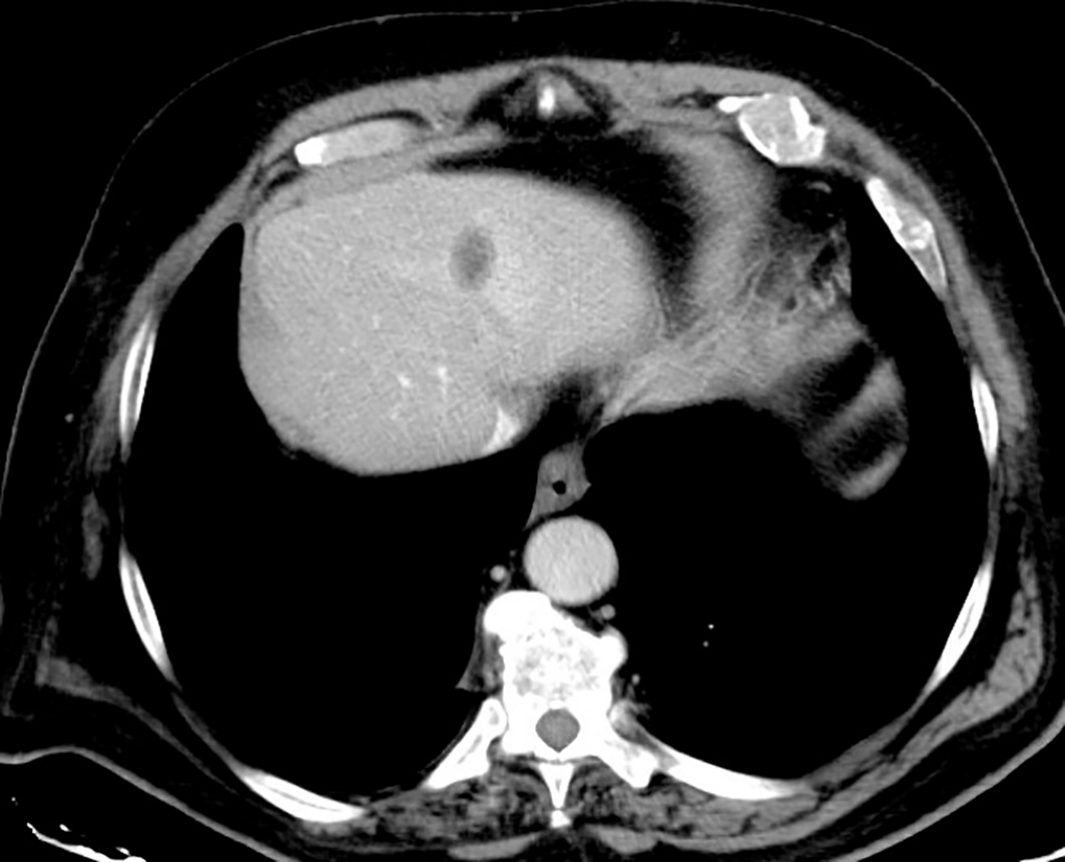
Hypodense area appears in the liver after irreversible electroporation (IRE). This low-density region represents the classical aspect of an electroporated parenchymal area.

RFA remains the most widely used thermo-ablative technique worldwide in this case scenario even though the first choice for the treatment of hepatic secondary lesions is still surgery and chemotherapy is considered a valid help to other, more effective, treatments (45) (**Figure 3**). The main concerns about RFA have focused on the high local recurrence rates, particularly in the treatment of masses larger than 3 cm in diameter, the potential incomplete tumour ablation near blood vessels because of the heat-sink effect, and the difficulty in US follow-up of RF lesions.

When it comes to hepatic tumours, the first indication for irreversible electroporation seems to be the treatment of metastatic lesions located in proximity to vital structures, like major bile ducts or large vessels, to spare them from thermal damage and to avoid the heat-sink effect at the same time. To date, IRE is only recommended for patients with a reasonable life expectancy or as a palliative treatment, even though preliminary studies have shown greater overall survivability if compared to chemotherapy alone (46). Metastasis size should not exceed 3.0 cm in order to avoid the chance of not obtaining a complete tumour ablation. As downsides, this procedure requires general anaesthesia and is therefore more expensive and potentially risky than the more traditional kind of ablation. An ablation technique such as IRE is needed, since its unique role in the treatment of recurrences located next to big vessels, and according to authors, more studies should be encouraged.

## Author contributions

All authors contributed to the article and approved the submitted version.

## Conflict of interest

The authors declare that the research was conducted in the absence of any commercial or financial relationships that could be construed as a potential conflict of interest.

## Publisher’s note

All claims expressed in this article are solely those of the authors and do not necessarily represent those of their affiliated organizations, or those of the publisher, the editors and the reviewers. Any product that may be evaluated in this article, or claim that may be made by its manufacturer, is not guaranteed or endorsed by the publisher.
